# Retinal dendritic cell recruitment, but not function, was inhibited in MyD88 and TRIF deficient mice

**DOI:** 10.1186/s12974-014-0143-1

**Published:** 2014-08-13

**Authors:** Neal D Heuss, Mark J Pierson, Kim Ramil C Montaniel, Scott W McPherson, Ute Lehmann, Stacy A Hussong, Deborah A Ferrington, Walter C Low, Dale S Gregerson

**Affiliations:** Department of Ophthalmology & Visual Neurosciences, University of Minnesota, Lions Research Bldg. Rm 314, 2001 6th St SE, Minneapolis, MN 55455 USA; Department of Neurosurgery, University of Minnesota, Minneapolis, MN 55455 USA

**Keywords:** Dendritic cells, Injury, Microglia, Retinal ganglion cells, NFκB

## Abstract

**Background:**

Immune system cells are known to affect loss of neurons due to injury or disease. Recruitment of immune cells following retinal/CNS injury has been shown to affect the health and survival of neurons in several models. We detected close, physical contact between dendritic cells and retinal ganglion cells following an optic nerve crush, and sought to understand the underlying mechanisms.

**Methods:**

CD11c-DTR/GFP mice producing a chimeric protein of diphtheria toxin receptor (DTR) and GFP from a transgenic CD11c promoter were used in conjunction with mice deficient in MyD88 and/or TRIF. Retinal ganglion cell injury was induced by an optic nerve crush, and the resulting interactions of the GFP^hi^ cells and retinal ganglion cells were examined.

**Results:**

Recruitment of GFP^hi^ dendritic cells to the retina was significantly compromised in MyD88 and TRIF knockout mice. GFP^hi^ dendritic cells played a significant role in clearing fluorescent-labeled retinal ganglion cells post-injury in the CD11c-DTR/GFP mice. In the TRIF and MyD88 deficient mice, the resting level of GFP^hi^ dendritic cells was lower, and their influx was reduced following the optic nerve crush injury. The reduction in GFP^hi^ dendritic cell numbers led to their replacement in the uptake of fluorescent-labeled debris by GFP^lo^ microglia/macrophages. Depletion of GFP^hi^ dendritic cells by treatment with diphtheria toxin also led to their displacement by GFP^lo^ microglia/macrophages, which then assumed close contact with the injured neurons.

**Conclusions:**

The contribution of recruited cells to the injury response was substantial, and regulated by MyD88 and TRIF. However, the presence of these adaptor proteins was not required for interaction with neurons, or the phagocytosis of debris. The data suggested a two-niche model in which resident microglia were maintained at a constant level post-optic nerve crush, while the injury-stimulated recruitment of dendritic cells and macrophages led to their transient appearance in numbers equivalent to or greater than the resident microglia.

**Electronic supplementary material:**

The online version of this article (doi:10.1186/s12974-014-0143-1) contains supplementary material, which is available to authorized users.

## Background

Neurodegenerative processes adversely affect vision in a significant portion of the human population, and are associated with glaucoma, age-related macular degeneration (AMD), diabetic retinopathy, ischemia, retinopathy of prematurity and traumatic injuries [1–4]. Irrespective of the cause of the degenerative process, it is evident that the immune system can be protective or pathogenic in neurodegeneration [5]. Studies show that microglia (MG) are not simply the scavengers of the nervous system; instead, they appear to play complicated, even contradictory, roles [6,7]. MG have been associated with clean-up of dead/dying neurons with minimal inflammation [8,9], restoration of health to damaged peripheral nervous system (PNS) neurons [10], and promotion of inflammation through secretion of proinflammatory molecules [11,12]. They also appear to kill neurons through effector mechanisms that include production of reactive oxygen species which disrupt axonal transport [13], and cytotoxic cytokines including TNF. FasL expression by MG may contribute to the death of Fas^+^ neurons [14,15].

Regardless of the injury, MG are rapid responders. Within minutes of a focal injury to brain or retina, neighboring MG extended processes to the site [16–18]. At later time points post-injury, other cells participate. The optic nerve crush (ONC) has been used as a model for neural injury, giving a discreet injury to a limited number of neurons, retinal ganglion cells, that progresses on a well-studied, consistent course [19,20]. By two to three days post-ONC, we found evidence that dendritic cells (DC) were recruited to the retina, increasingly associated with the ganglion cell layer (RGC) and the nerve fiber layer (NFL) of the retina, and increased in number for approximately ten days, equaling the MG in total number per retina [21]. The number of DC remained elevated for more than two months, gradually declining in number. Many DC were closely associated with the axons of RGC post-ONC [21]. DC dominated this close interaction for at least three weeks post-injury, raising the question of whether their response was protective, harmful, or unrelated to survival of RGC.

The well-known function of DC as antigen presenting cells may be important, consistent with reports that suggest that adaptive immunity mediated by T cells may be neuroprotective [22–25]. However, very few lymphocytes were found after this sterile injury, suggesting that the activity of these DC may not be limited to antigen presentation. We present evidence that DC are active participants in the injury response. Analysis of DC from MyD88 and TRIF single and double knockout mice showed that DC in the knockout mice were much less efficiently recruited to the retina post-injury, but the smaller number that was present remained fully able to take up DiI^+^ debris via phagocytosis of DiI-labeled RGC and axon debris after an ONC. MG compensated for the reduced number of DC in MyD88 and/or TRIF deficient mice by increasing their uptake of DiI^+^ debris.

## Materials and methods

### Animals

CD11c-DTR/GFP mice (CDG) mice on the B6 background (B6.FVB-Tg(Itgax-DTR/EGFP)57Lan/J), express a chimeric protein comprised of GFP and the diphtheria toxin receptor (DTR) under control of the CD11c promoter [26]. Wild type (wt) C57BL/6J (B6) mice were obtained from Jackson Laboratory (Bar Harbor, ME, USA). MyD88/TRIF double knockout mice (MTdko) on the B6 background were bred from pairs obtained from Dr. Stephen Jameson, University of Minnesota. The double knockout mice were backcrossed to B6 mice to generate the single knockout mice, MyD88-deficient (Mko) mice and TRIF-deficient (Tko) mice. The single and double knockout mice were backcrossed to CDG mice to allow visualization of the GFP reporter for CD11c. All mice were CD45.2. Transgenic mice were bred in house. All mice were *rd8* negative. Mice were handled in accordance with the Association for Research in Vision and Ophthalmology Statement for the Use of Animals in Ophthalmic and Vision Research and University of Minnesota Institutional Animal Care and Use Committee guidelines.

### Optic nerve crush

The optic nerve crush (ONC) was performed as described [21,27], except for use of #2197E DSAEK forceps (Ambler Surgical Corp., Exton, PA, USA) for the crush procedure. Briefly, the optic nerve was clamped for three seconds at a point 1 to 2 mm from the posterior pole of the globe.

### RGC labeling

Injection of fluorescent dye, either Fluorogold (FG) or di-alkyl-indocarbocyanine (DiI), into the superior colliculus was done to retrogradely label the RGC. Manipulations were done in a stereotactic device. A midline incision was made in the scalp to expose the skull. A unilateral 1 mm hole was drilled at –3.5 mm from bregma and +1.2 mm from midline. A 10-μl syringe and non-coring needle attached to a micromanipulator was inserted to a depth of −2.5 mm from the surface of the brain. Four percent dye in 1.5 μl of saline was injected over the course of 2 minutes. After slow removal of the syringe, the scalp was sutured with 4-0 silk. FG was used to count surviving RGC and was administered after the ONC four days before retina harvest. The FG diffuses rapidly to the opposite hemisphere of the brain, so that the RGC of both retinas become equivalently stained even if the dye was injected unilaterally in the brain. DiI was used to label the RGC with red fluorescence for experiments to detect labeled RGC debris in the mononuclear cells of the retina by flow cytometry and fluorescence microscopy. DiI was administered seven to ten days before the ONC.

### Flow cytometry of retinal cells

Mice were euthanized, perfused, and the retinas removed as described [21]. Retinas were dissociated in 0.5 μg/ml Liberase/Blendzyme3 (Roche, Indianapolis, IN, USA) and 0.01% DNase in Dulbecco’s phosphate-buffered saline (DPBS), stained with indicated antibodies, and analyzed as described [21,28,29]. Analyses were based on the examination of all immune cells collected from one or more retinas, as specified.

### Immunostaining of retinal flatmounts

Retinal flatmounts were prepared, stained, and analyzed as described [21]. Primary antibodies included: rat anti-CD11b to stain myeloid cells (clone M1/70, BD Biosciences, San Jose, CA, USA) and anti-β3-tubulin to stain RGC and their axons (clone TU-20). Secondary antibodies (Invitrogen, Life Technologies, Grand Island, NY, USA) included: Alexa Fluor 594 donkey anti-rat IgG for anti-CD11b; and Alexa Fluor 405 rabbit anti-mouse IgG for anti-β3 tubulin. Cell nuclei were stained with 4′,6-diamidino-2-phenylindole (DAPI, Vector Laboratories, Burlingame, CA, USA).

### TUNEL-stained retinal sections

Eyes were enucleated and immediately snap-frozen in Tragacanth (Sigma, St. Louis, MO, USA). Retinas were sectioned (12 μm) through the optic nerve. Detection of apoptotic nuclei was accomplished by terminal deoxynucleotidyl transferase-mediated dUTP nick-end labeling (TUNEL) using the *In Situ* Cell Death Detection Kit, Fluorescein (Roche, Indianapolis, IN, USA). Slides were cover slipped with VECTASHIELD Mounting Medium containing DAPI (Vector Laboratories, Burlingame, CA, USA) to visualize the nuclei.

### Retinal morphology measurements

The density of nuclei in the ganglion cell layer (GCL) was measured on DAPI-stained retinal sections. For each retinal section, three images were taken on either side of the optic nerve at 500 μm intervals. The length of the GCL was measured using BIOQUANT NOVA PRIME 6.90.10 (BIOQUANT Image Analysis, Nashville, TN, USA). The number of DAPI-stained nuclei in the GCL was counted from these images and normalized to the length of the retinal section to calculate the nuclei density (nuclei/μm). The same retinal sections were also stained with TUNEL. The TUNEL^+^ nuclei in these same sections were counted and cross-referenced with the DAPI stained nuclei to ensure presence of a nucleus.

### Statistics

Data, whether expressed numerically or graphically as a mean, included the standard deviation; standard error was not used. Data analyses for significance were done with the InStat3 package from GraphPad Software (San Diego, CA, USA). Comparisons of three or more data sets were done with one way analysis of variance (ANOVA) using the Dunnett multiple comparisons test with a designated control set. Comparisons of two data sets were done with a two-tailed, unpaired *t*-test.

## Results

### Optic nerve crush injury

**Figure 1:**
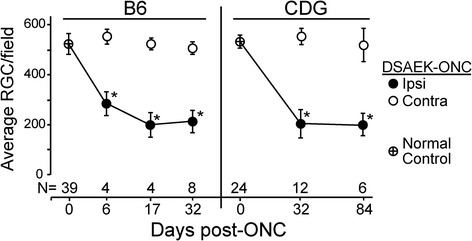
**CDG and B6 retinal ganglion cells (RGC) survive similarly after an optic nerve crush (ONC).** RGC survive in greater numbers using DSAEK forceps for the ONC. *All post-ONC counts of RGC differed from normal controls, as well as the contralateral RGC, *P* < 0.05. Normal control RGC counts represent the RGC counts of both retinas after unilateral injection into the superior colliculus. Ipsilateral - manipulated side; contralateral - opposite, unmanipulated side. Counts are average RGC numbers/retinal field where each retina count is the average of eight fields/retina, ± SD. N = number of mice. For example, an N of 4 represents 32 fields. Field size = 0.190 mm^2^.

The ONC procedure yielded a significant, reproducible sterile injury to the RGC (Figure 1), providing the opportunity to study factors that may affect recruitment of mononuclear cells and their interactions with the injured RGC. Survival of RGC from wt B6 mice and CDG mice was similar (Figure 1). The autocount procedure and results are described in the Additional file 1: Figure S1. Extrapolating our counts/field to the entire normal C57BL/6J retina yielded a total of 46,324 ± 1,968 RGC, which compares well with values from other reports obtained by counting axons (44,860 [30] and 47,113 [31]) or retrograde labeled RGC (49,823 [32]).

### Identification of retinal DC and MG/macrophages

**Figure 2:**
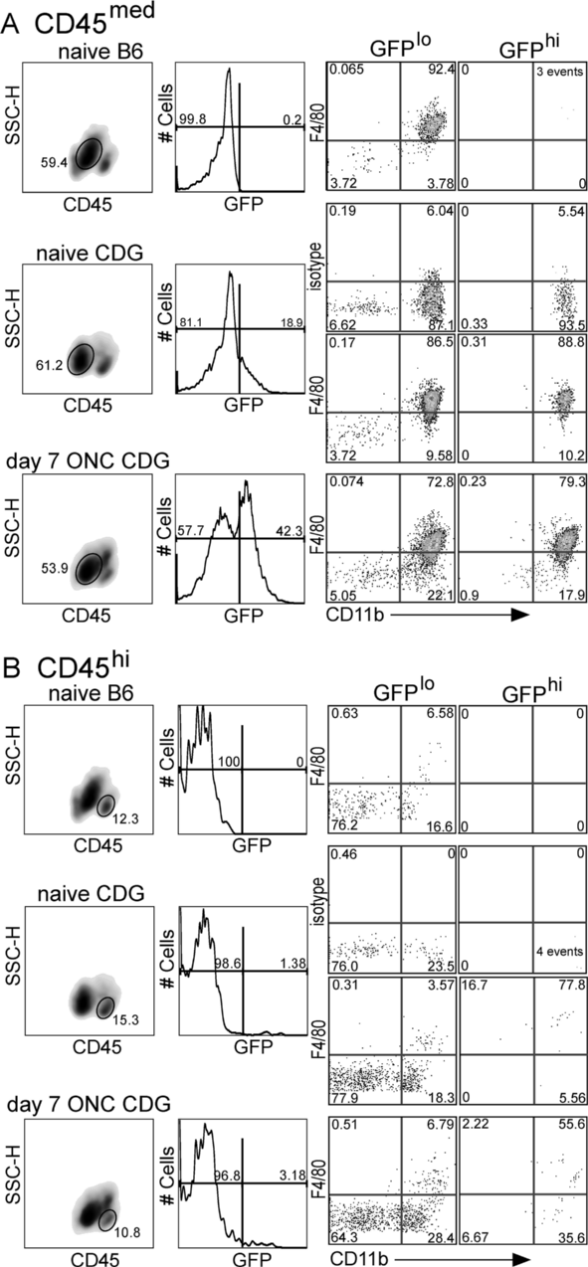
**Retinal GFP** ^**hi**^ **cells are CD11b** ^**+**^ **and express F4/80.** The GFP^hi^ cells in the retina of CDG mice are found in the CD45^med^ region associated with microglia (MG), and in the CD45^hi^ populations. **(A)**, Analysis of F4/80 and GFP expression in retinal CD45^med^ cells from naive B6, naive CDG, and CDG mice post-ONC. **(B)**, Expression of GFP and F4/80 in CD45^hi^ cells from the same retinas shown in **(A)**. The quadrants are labeled with the percent of cells contained in each quadrant.

We previously showed that the CD11b^+^GFP^hi^ cells in quiescent CDG retina were morphologically similar to MG [21]. Flow cytometry showed that the CD11b^+^GFP^lo^ and CD11b^+^GFP^hi^ DC in the CD45^med^ region commonly associated with MG were indistinguishable by several parameters, including expression of F4/80, whether from quiescent or post-ONC retinas (Figure 2A). The CD45^hi^ region also contains a small number of CD11b^+^ cells, most are F4/80^+^ (Figure 2B). The influx of GFP^hi^ cells post-ONC was easily detected by sequentially gating on CD45^+^ cells, and then confirming that the GFP^hi^ cells expressed CD11b. At 7 days post-crush, the GFP^hi^ cells accounted for approximately 40% of the total CD45^+^11b^+^ cells. The CD11b^+^GFP^hi^ cells do not express detectable levels of Ly6G (clone 1A8) or Ly6C/G (Gr-1, clone RB6-8C5 (data not shown)), showing that neutrophils did not contribute to the counts. These properties allowed identifying these cells in four groups (Table 1) for the studies that follow.

**Table 1:** **Phenotype of the GFP** ^**hi**^ **DC and GFP** ^**lo**^ **mononuclear cells from retina**

**CDG retinal cells**	**GFP** ^**a**^	**CD45** ^**a**^	**CD11b** ^**a**^	**F4/80** ^**a**^
GFP^hi^ DC (CD45^med^)	hi	med	hi	+
GFP^hi^ DC (CD45^hi^)	hi	hi	hi	+
MG/monocyte/macrophage (CD45^med^)	lo^b^	med	hi	+
monocyte/macrophage (CD45^hi^)	lo	hi	hi	+

^a^Flow cytometry.

^b^Resistant to DTx depletion in CDG mice.

### Retinal DC associate with RGC axons and soma after an ONC

**Figure 3:**
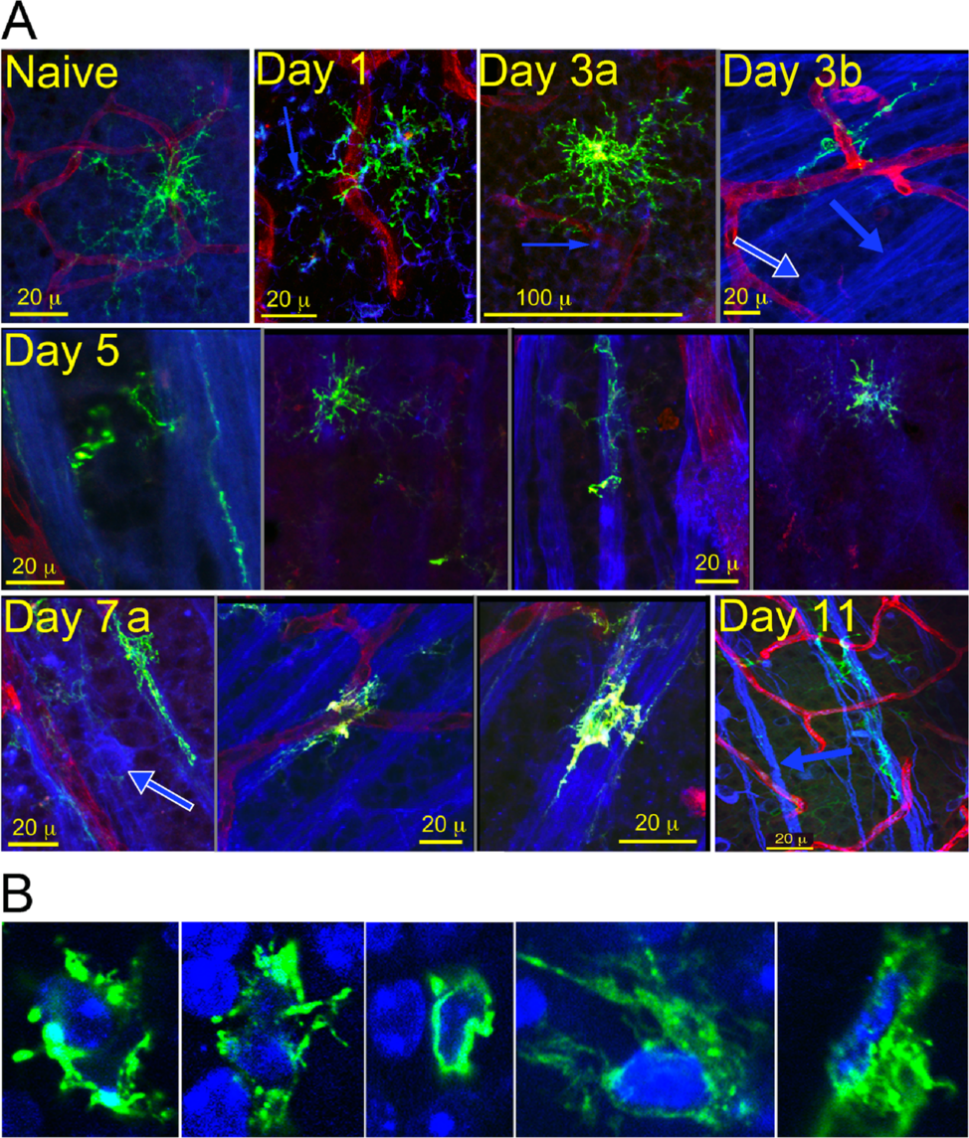
**Recruitment, redistribution and interaction of retinal GFP** ^**hi**^ **DC with retinal ganglion cells (RGC) and their axons following an optic nerve crush (ONC) injury. (A)**, GFP^hi^ DC in naive retina, and at 1, 3, 5, 7, and 11 days post-ONC. GFP^hi^ DC, green; isolectin B4-stained blood vessels, red; anti-β3 tubulin stained RGC somata (blue/white arrows), nerve fibers (large blue arrows), and dendrites (small blue arrows). **(B)**, GFP^hi^ DC surround and engulf RGC somata ten days post-ONC. DAPI, blue; GFP^hi^ DC, green.

In quiescent retina, a small number of ramified GFP^hi^ DC were distributed in the inner plexiform layer (IPL) immediately below the soma of the RGC (Figure 3A). This morphology was unchanged at day 1 post-ONC (Figure 3A). DC in close association with injured RGC were first detected three days post-ONC, when a careful search of three retinas found a few GFP^hi^ DC closely associated with RGC nerve fibers (Figure 3A). Dendrites (small blue arrows) show GFP^hi^ DC in the IPL at day 1 and day 3a. Large blue arrows show axons bundled into nerve fibers (day 3b). Somata (white/blue arrows) are shown in days 3b and 7a. By five days post-ONC, numerous close associations between the GFP^hi^ DC and nerve fibers were found (Figure 3A). Counts of representative 20X fields from three retinas harvested at day 5 post-ONC showed that 16% of the total GFP^hi^ DC in retina were closely associated with nerve fibers or soma. Close association of the GFP^hi^ DC with the NFL peaked at 7 to 11 days (Figure 3A). We previously showed that GFP^hi^ DC dominated the interaction of CD11b^+^ cell populations with the NFL at seven days post-ONC [21]. By 11 days post-ONC, the density of β3-tubulin^+^ fibers in the NFL was reduced, but GFP^hi^ DC were still found in close contact with remaining fibers (Figure 3A). At five or more days post-ONC, DC were found in contact with the RGC soma, and these appeared to be engulfing the RGC (Figure 3B). The close association of the DC with the RGC and their axons raised questions about the molecular basis for these interactions that may affect RGC survival following an ONC.

### Roles of DC versus MG/macrophages in clearance of damaged RGC

**Figure 4:**
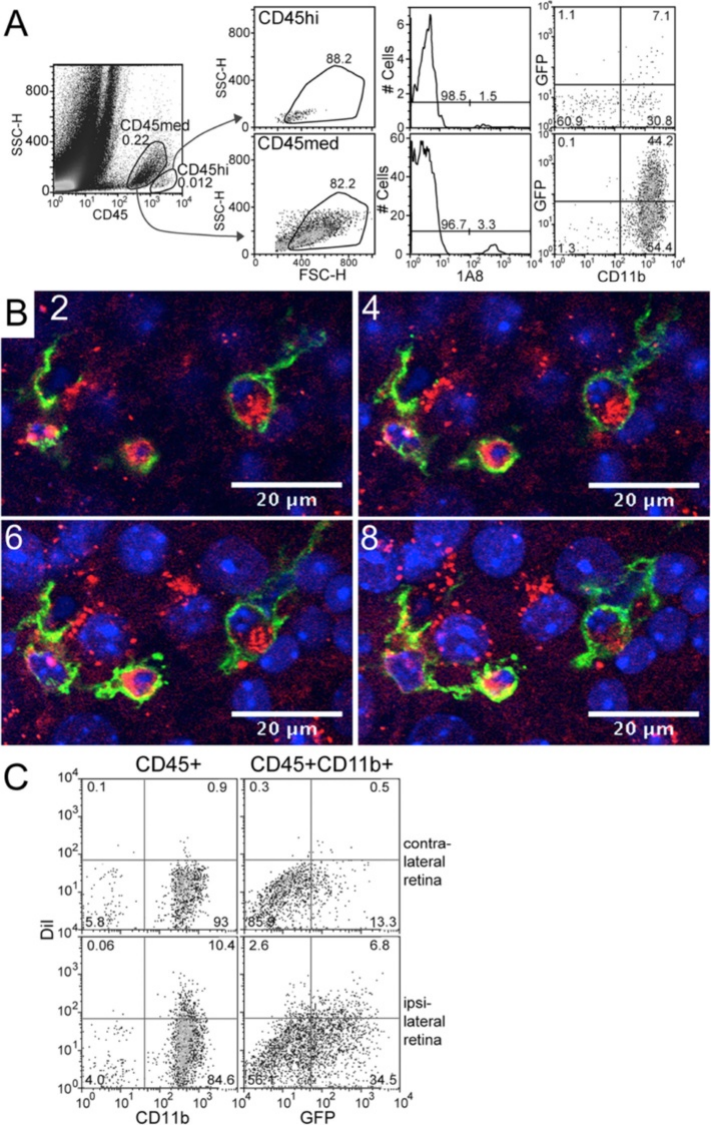
**Detection of retinal ganglion cell (RGC) phagocytosis by GFP** ^**hi**^ **and GFP** ^**lo**^ **CD11b** ^**+**^ **cells in CDG retina post-ONC. (A)** Depiction of CD45^hi^ and CD45^med^ gating, elimination of 1A8^+^ PMN, and selection for CD11b^+^ cells that are GFP^lo^ or GFP^hi^. **(B)** Sequential confocal sections of GFP^hi^ DC engulfing DiI-labeled RGC seven days post-ONC. DiI-labeled RGC, red; GFP^hi^ DC, green; DAPI-stained nuclei, blue. **(C)** Detection and quantitation of DiI-labeled CD45^med^CD11b^+^GFP^hi^ DC and GFP^lo^ cells by flow cytometry. Ipsilateral retinas were labeled by injection of DiI into the superior colliculus. After seven days, mice were given an ONC; retinas were harvested six days later.

Candidate phagocytic cells in the retina that may clear damaged RGC are CD45^+^CD11b^+^Ly6G^−^ mononuclear cells. To detect the relative contributions of MG, recruited macrophages, and recruited GFP^hi^ DC to the clearance of injured RGC post-ONC, the RGC were pre-labeled with DiI to detect CD45^+^CD11b^+^ cells that had phagocytosed DiI-labeled RGC. The CD11b^+^ phagocytic cells in the retina are found in two populations based on CD45 staining intensity, CD45^hi^ and CD45^med^ (Figure 4A, Table 1). The CD45^hi^ population of CDG retina contains CD11b^+^ cells that include GFP^hi^ DC, GFP^lo^ monocytes, macrophages, and polymorphonuclear granulocytes (PMN). The PMN were excluded by staining with clone 1A8, which identifies Ly6G^+^ PMN. Although PMN are recruited onto the retinal surface at early time points post-ONC, a PMN in contact with the NFL or RGC was not observed (data not shown), and they were routinely excluded from further analysis. The CD45^med^ population post-ONC includes MG, recruited macrophages and GFP^hi^ DC (Figure 4A, Table 1).

As shown by others, and in Figure 4B, RGC were well-labeled seven days after injection of DiI into the superior colliculus [33]. After seven days DiI labeling, mice were given an ONC. Seven days post-ONC, DiI^+^ RGC were being engulfed by GFP^hi^ DC, as shown in a series of 1-μm optical sections from confocal microscopy (Figure 4B). Slices 2, 4, 6 and 8 show the association of the DC with individual RGC somata at 2-μm increments. Further verification that CD11b^+^ cells would take up DiI-labeled cellular debris after an ONC was found by flow cytometric analysis of retinas after seven days DiI labeling followed by an ONC. The retinas were harvested at day 6 post-ONC. DiI labeling was found in both the GFP^hi^ DC and GFP^lo^ MG/macrophages from the CD45^med^ population (Figure 4C). This gating strategy was used for further analysis of DiI^+^ DC and macrophages.

**Figure 5:**
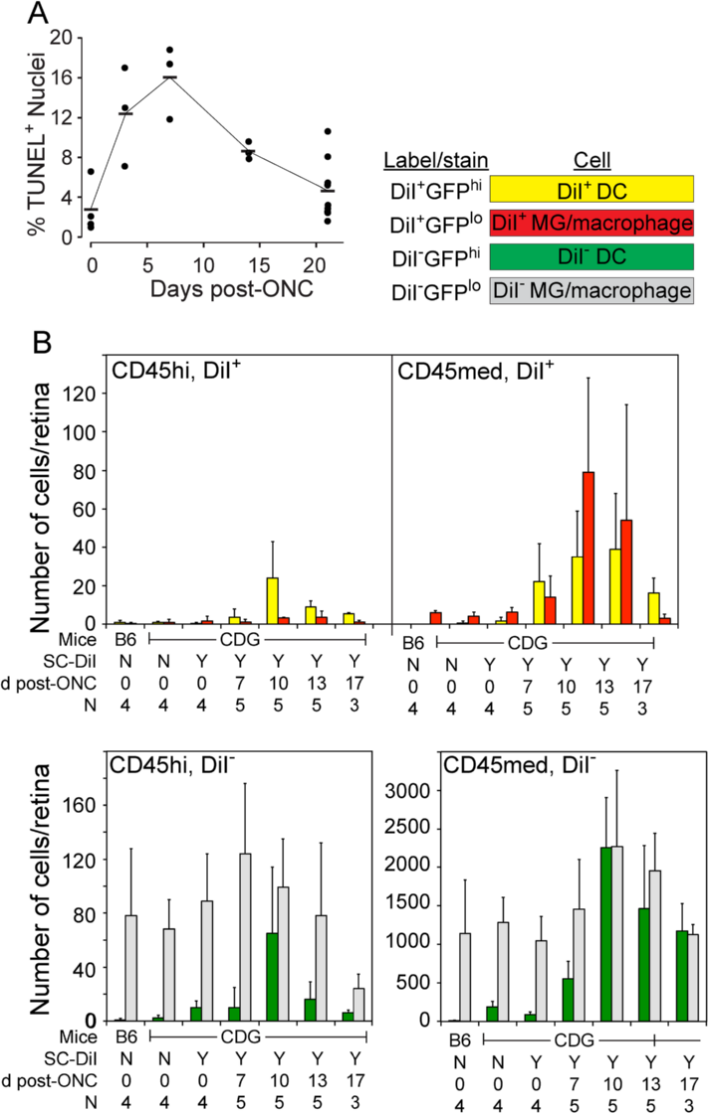
**Phagocytosis of retinal ganglion cells (RGC) following an optic nerve injury (ONC). (A)** Time course of RGC apoptosis following an ONC. **(B)** Analysis of DiI uptake in GFP^hi^ DC and GFP^lo^ MG/macrophages following an ONC. Retinas were labeled by injection of DiI into the superior colliculus. After 7 days the mice were given an ONC; retinas were harvested 7, 10, 13, and 17 days later. CD45^+^ cells were examined by flow cytometry as shown in Figure 4. All cells in **(B)** are CD11b^+^.

The time course of DiI uptake into CD11b^+^ cells was determined next. Based on the progression of RGC apoptosis, estimated by counts of TUNEL^+^ cells in the RGC layer (Figure 5A), DiI-labeled retinas were harvested from 7 to 17 days post-ONC and analyzed by flow cytometry to detect DiI uptake by phagocytic CD11b^+^ cells in CD45^med^ and CD45^hi^ populations (Figure 5B). The number of DiI^+^CD11b^+^ cells peaked at ten days post-ONC in both CD45^hi^ and CD45^med^ populations. Although the total number of CD11b^+^ cells in the CD45^hi^ population was much lower than the CD45^med^ population, the frequency of CD45^hi^DiI^+^ cells at day 10 post-ONC was much higher (14%, 27 of 191 cells), than that found in the CD45^med^ population (2.5%, 115 of 4,638 cells) (Figure 5B). Of the total CD45^hi^DiI^+^CD11b^+^ cells, 89% were GFP^hi^, whereas only 35% of the CD45^med^DiI^+^ cells were GFP^hi^. These results showed that mononuclear cells were actively phagocytosing DiI-labeled RGC, and proportionately more of the DiI^+^ cells were CD45^hi^ DC. Since day 10 post-ONC was the peak of DiI uptake, day 10 was used for subsequent experiments.

### MyD88/TRIF and recruitment of CD11b^+^ myeloid cells to retina post-ONC

**Figure 6:**
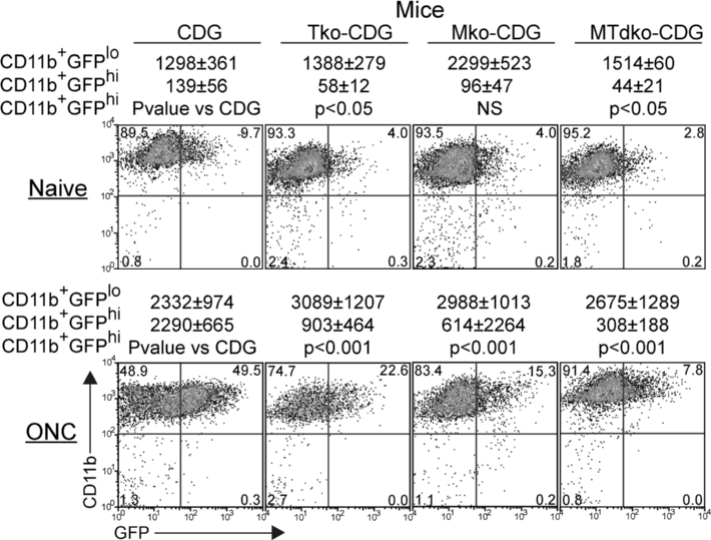
**Recruitment of CD45** ^**med**^ **GFP** ^**hi**^ **cells to retina in Tko, Mko, or MTdko mice on the CDG background.** Retinas were harvested ten days after an ONC. Numbers in the quadrants are percents, and are the averages of at least four samples. *P*-values for differences in the populations are shown.

**Figure 7:**
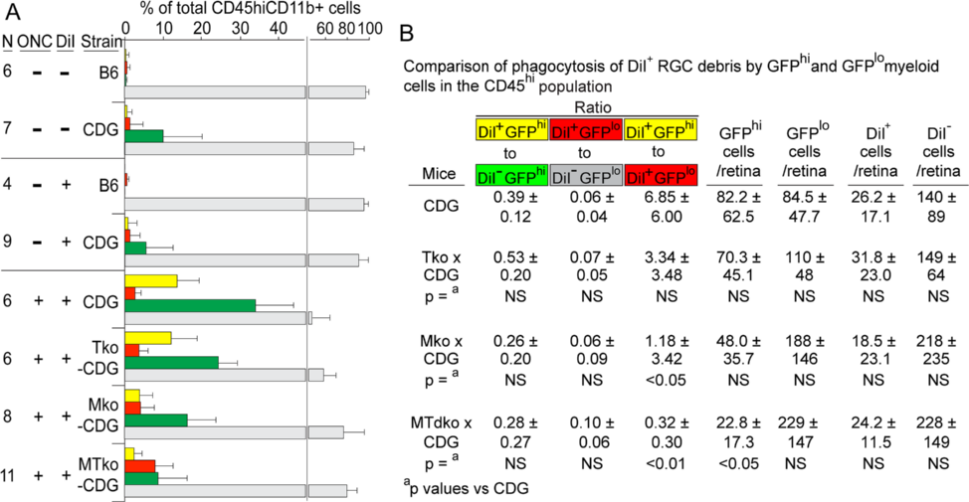
**DiI uptake by CD45** ^**hi**^ **cells. (A)** Flow cytometric analysis of DiI uptake by GFP^hi^ and GFP^lo^ cells in the CD45^hi^ population of retina from the indicated strains harvested after retrograde labeling of RGC with DiI. The retina was DiI labeled by injection of DiI into the superior colliculus seven days prior to the ONC. Retina was harvested ten days post-ONC where indicated. **(B)** Summary of cell counts and *P*-values in mice receiving DiI and an ONC.

**Figure 8:**
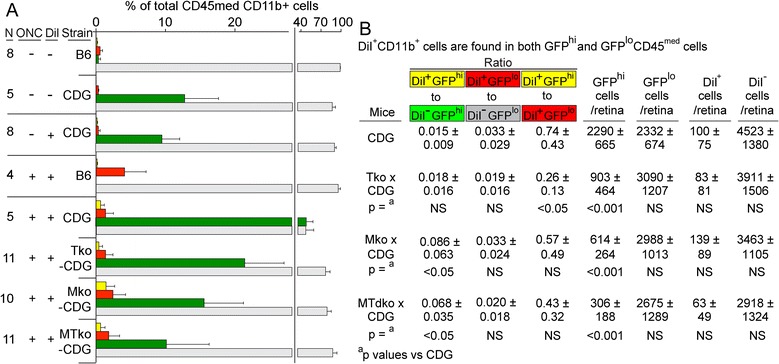
**DiI uptake by CD45** ^**med**^ **cells. (A)** Flow cytometric analysis of the uptake of DiI by GFP^hi^ and GFP^lo^ cells in the CD45^med^ population of retina from the indicated strains harvested after retrograde labeling of RGC with DiI via injection of DiI into the superior colliculus seven days prior to the ONC. Retina was harvested ten days post-ONC. Application of the DiI label, and performance of the ONC were as indicated on the axis. **(B)** Summary of cell counts and *P*-values.

Given the active participation of mononuclear cells in the clearing of RGC debris, we sought evidence for the contribution of toll-like receptors (TLR) to this response. A number of recent reports suggested roles for TLR in the responses of CNS tissue to injury and/or neurodegeneration [34,35]. The adaptor proteins MyD88 and TRIF link TLR ligation to NFκB activation. Their deletion has been reported to reduce neural inflammatory responses to infectious agents [36–39], and has been associated with an increase in neural deficits or diminished neurotoxicity [40]. Since the GFP^+^ DC appeared in substantial numbers in the retina following injury, were closely associated with injured neurons, and phagocytosed RGC and axon debris, we asked if these activities correlated with TLR-mediated sensing of the local environment. Mko, Tko, and MTdko mice were backcrossed to the CDG background to examine the effects of eliminating these proteins on the influx of GFP^hi^ DC and GFP^lo^ MG and macrophages in response to the ONC. Prior to injury, Tko and MTdko mice had reduced numbers of CD45^med^GFP^hi^ DC in the retina compared to control mice (Figure 6, Top). Deficiencies in either MyD88 or TRIF or both also led to a substantial reduction in the number of GFP^hi^ DC that were recruited in response to an ONC (Figure 6, Bottom). The double knockout mice gave the greatest decline in retinal GFP^hi^ DC for both basal and post-ONC conditions, suggesting that the NFκB pathway regulated the response of mononuclear cells to an ONC. The flow cytometry conditions shown in Figures 4 and 6 were then analyzed for DiI to generate the data for Figures 7 and 8, which follow.

### MyD88/TRIF deficiency diminished uptake of DiI-labeled RGC debris by GFP^hi^ DC post-ONC

The CD45^hi^ and CD45^med^ populations were examined for the effects of MyD88 and/or TRIF deficiency on clearance of DiI-labeled RGC debris. The mononuclear cells in the CD45^hi^ population became labeled with DiI after an ONC, regardless of the presence or absence of MyD88 or TRIF (Figure 7); the differences due to MyD88 and TRIF activity were found in the number of cells that were recruited by the injury. In control CDG mice, 39% of the GFP^hi^ DC were DiI labeled (yellow versus green bars), compared to 6% of CD11b^+^GFP^lo^ cells that were found to be DiI^+^ (red versus gray bars) (Figure 7B). Although the recruitment of GFP^hi^ DC into the CD45^hi^ population declined in the MTdko mice, the fraction of DiI^+^GFP^hi^ cells relative to GFP^+^ cells did not change significantly (Figure 7B). The fraction of DiI^+^GFP^lo^ cells ranged from 6 to 10%, and was not significantly different. Although the total of CD45^hi^GFP^hi^ cells declined substantially (from 82 to 23), the total number of DiI^+^ cells was not different (Figure 7B); the GFP^lo^ cells appeared to compensate for the smaller number of GFP^hi^ DC in the uptake of DiI-labeled debris. Clearly, the DiI^+^GFP^hi^ DC and the DiI^+^GFP^lo^ cells responded differently to the deficiencies in MyD88 and/or TRIF.

As shown above, CD11b^+^DiI^+^ cells were also found in the CD45^med^ population, but their frequency was much lower than seen in the CD45^hi^ populations (Figure 8A). The effect of MyD88/TRIF deficiency differed, relative to the results seen above for CD45^hi^ cells. For example, the number of DiI^+^ cells, relative to the total number of CD11b^+^ cells, was constant in control versus MTdko mice (1:46 in CDG retina versus 1:47 in CDG x MTdko retina) (Figure 8B). Although the total number of cells in the CD45^med^ population of CDG retina was approximately 27-fold higher than in the CD45^hi^ population, the total number of CD45^med^DiI^+^ cells was only 4-fold higher. A similar comparison of these populations in MTdko retina showed an 11-fold difference in total cell number and a 2.5-fold difference in DiI^+^ cells.

The basis for maintaining the DiI uptake in the face of a substantial decrease in GFP^hi^ DC is the significant shift in the distribution of DiI^+^ cells. Their frequency in GFP^hi^ cells from CDG x MTdko retina was higher than in GFP^hi^ cells from CDG retina (0.015 in CDG retina versus 0.068 in CDG x MTdko retina; *P* < 0.05). Conversely, the proportion of DiI^+^GFP^lo^ cells was not different in the CDG versus CDG x MTdko retina (0.020 versus 0.033, NS) (Figure 8B). Since the number of GFP^hi^ DC/retina substantially declined in the MTdko mice (*P* < 0.001), but the DiI^+^ cells did not, showed that uptake of debris was not significantly compromised by MyD88 and/or TRIF deficiency. The effect of MyD88/TRIF deficiency was more closely associated with recruitment of GFP^hi^ cells. The GFP^hi^ DC clear debris with similar efficiency, even though their numbers declined.

### Depletion of GFP^hi^ cells from retina by systemic DTx reveals their replacement with GFP^lo^ cells

The results above revealed an altered response to injured RGC following an ONC in MyD88 and/or TRIF deficient mice. The GFP^hi^ DC population was most actively associated with injured RGC, and most affected by manipulations of MyD88 and TRIF. Since they are DTx sensitive, DTx treatment of the mice allowed studies of the participation of DC in RGC degeneration post-ONC, within the context of MyD88 and TRIF deficiencies. Several DTx treatment protocols were examined to confirm the DTx sensitivity of retinal DC, and devise DTx treatment protocols that would allow comparison of the ONC injury response in the presence or absence of retinal DC.

**Figure 9:**
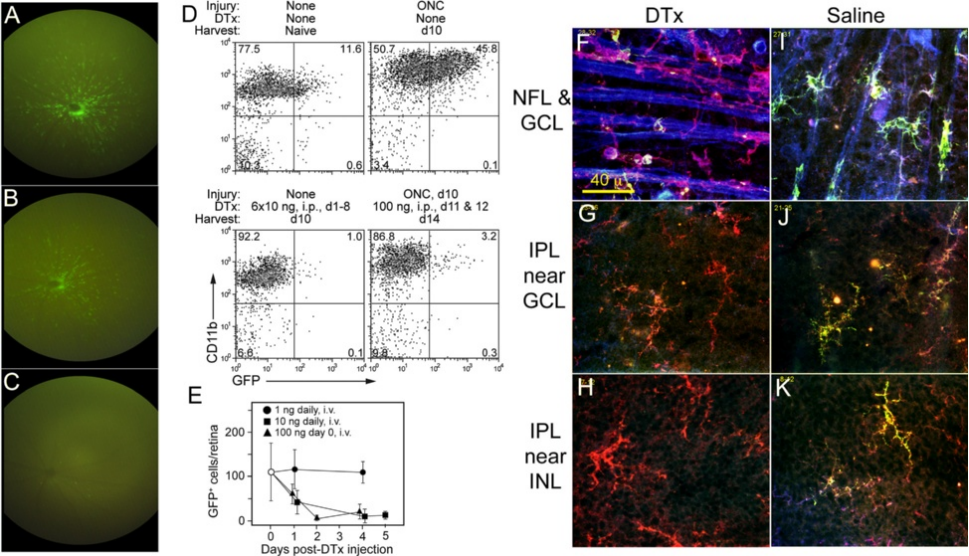
**Systemic administration of DTx to the CD11c-DTR/GFP mice leads to depletion of the GFP** ^**hi**^ **DC. (A-C)** Serial fundus photographs of the same retina following an ONC and DTx treatment via ip injection. **(A)** GFP^hi^ DC at 12 days post-ONC. **(B)** Fundus photograph of retina one day after ip injection of 200 ng DTx. **(C)** Photograph of retina two days post-ip DTx injection. **(D)** Flow cytometry of retina following ip injection of DTx under two different protocols; six daily injections of 10 ng into a naive mouse, and 2 injections of 100 ng into a mouse at days 11 and 12 post-ONC. **(E)** Daily serial injections of 10 ng DTx in naive mice. **(F**-**K)** Confocal microscopy of retinal flatmounts in ONC-injured retinas. **(F-H)** DTx (200 ng) was injected ip 48 hours prior to harvest, at 15 days post-ONC. **(F)** Cells and nerve fibers in the NFL post-DTx. **(G-H)** Cells at two levels in the IPL. All images in each vertical column are from the same confocal field at different depths into the retina. **(I-K)** Saline, 1 μl, was injected ip 48 hours prior to harvest, at 15 days post-ONC. **(I)** Cells and nerve fibers in the NFL. **(J-K)** Cells at two levels in the IPL. GFP^lo^CD11b^+^ cells stained red; GFP^hi^CD11b^+^ cells stained yellow-green. NFL, nerve fiber layer; IPL, inner plexiform layer at two depths is shown, immediately below the retinal ganglion cell layer (RGC) soma, and adjacent to the INL.

To explore differences between GFP^hi^ DC versus GFP^lo^ macrophages with respect to the close association with RGC axons in the NFL post-ONC, systemic injections of DTx were examined. The ip (intraperitoneal) route was chosen to facilitate *in vivo* imaging, since the optical properties of the cornea would be preserved, and to establish that DTx readily crossed the blood/retinal barrier. Serial fundus photographs showed that DTx treatment via ip injection effectively depleted GFP^hi^ DC recruited to the retina by an ONC (Figure 9A-C). Panel A showed an elevated number of GFP^hi^ DC at 12 days post-ONC. Panel B showed noticeable depletion in the same retina one day after ip injection of 200 ng DTx. Panel C revealed that two days post-DTx injection gave near-total depletion of GFP^hi^ cells. Six serial ip injections of 10 ng DTx, or two ip injections of 100 ng, substantially depleted the GFP^hi^ DC from uninjured versus crush-injured retinas, respectively, based on flow cytometry (Figure 9D). Importantly, both showed retention of GFP^lo^CD11b^+^ cells representing the MG/macrophages. Direct counts of GFP^hi^ cells in retinal flatmounts confirmed their depletion (Figure 9E). However, repeated systemic use of DTx for more than one week, even at low doses, was toxic. Accordingly, the short-term, high-dose protocol was used to explore the observation above that the MG replaced the DC interaction with RGC and axons, physically and functionally, in the MTdko mice.

Analysis of DTx depletion of GFP^hi^ DC by confocal microscopy of retinal wholemounts of ONC-injured CDG retinas given saline or 200 ng DTx ip showed depletion of the GFP^hi^ DC from the nerve fibers of the RGC (Figure 9F-K). In the saline-treated control mouse, multiple GFP^hi^ DC in close contact with the nerve fibers were found, and only a single GFP^lo^ cell can be seen (Figure 9I). GFP^hi^ DC were prominent in the underlying IPL (Figure 9J and K). We previously showed that at 18 hours post-DTx, the retinal GFP^hi^ DC were depleted and the axons showed no association with GFP^hi^ cells or with GFP^lo^CD11b^+^ MG/macrophages. Instead, the MG/macrophages were seen intact, in the same field, below the RGC/NFL [21]. We show here that extending the time after DTx treatment to 48 hours before harvest revealed replacement of the GFP^hi^ DC by the GFP^lo^ macrophages/MG on the nerve fibers (Figure 9F), and continued presence of GFP^lo^ MG/macrophages in the underlying IPL (Figure 9G and H).

### A two-niche model

**Figure 10:**
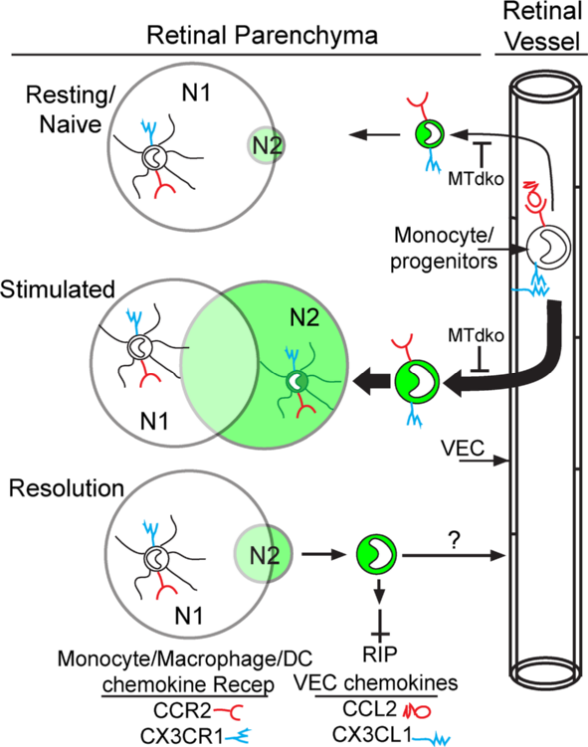
**A two-niche model for resident and recruited cells in retina post-injury.** The sizes of the niches, N1 (MG/macrophage niche) and N2 (GFP^hi^ DC niche), as well as the arrows denoting pathways, were drawn to suggest relative sizes, and changes due to injury. VEC, vascular endothelial cells.

Taken together, the data supports a two-niche model for resident and recruited cells in retina (Figure 10). Niche 1 (N1) is occupied by MG/macrophages. In the resting retina, this population far outweighs the GFP^+^ DC in niche 2 (N2). Upon stimulation, the majority of cells occupy an expanding N2 population. Shrinkage of N2 occurs with resolution, although levels remain somewhat elevated over time. Evidence from the MTdko mice suggests a role for NFκB signaling in the migration of cells into the retina under both resting and stimulated conditions.

## Discussion

We previously reported that DC identified as CD11b^+^GFP^hi^ cells in CDG mice strongly responded to retinal injury, and that their numbers often equaled those of the MG, or other recruited GFP^lo^ macrophages [21]. DC are central components in innate and adaptive immunity, expressing a wide range of receptors that sample the environment for molecules indicative of cell health, infection or disease. Their extensive and close association with RGC and their axons after an ONC raised the possibility that they were active participants in the RGC injury response. TLR, and several other receptors, are important sensors linked to NFκB via the adaptor proteins MyD88 and TRIF [41]. Some studies have shown that neural inflammation was reduced, and neuron survival enhanced following injuries in MyD88- or TRIF-deficient mice [42–45], while others reported that MyD88 deficiency led to exacerbation of the injury response and less neuroprotection [46]. We explored the retinal DC response in mice deficient in one or both adaptor proteins.

Unlike MG, DC were transient participants in the injury response, first observed interacting with the NFL at three days post-injury, rising rapidly to peak at seven to thirteen days post-injury, and then declining in number. The subpopulation of retinal GFP^hi^ DC that expressed CD45 at a high level, resembling that of circulating myeloid DC progenitors, was most active in acquiring the DiI label contained in the RGC. GFP^hi^ cells engulfing DiI-labeled RGC soma post-ONC were readily identified by fluorescence microscopy. GFP^hi^ DC with CD45 expression at a lower level similar to MG also took up DiI post-injury, as did MG/macrophages that were CD45^med^ and GFP^lo^.

A key difference between GFP^hi^ DC and MG is that relatively few GFP^hi^ DC remain in the retina long-term, whereas MG are defined as long-term resident macrophages. Emigration of GFP^hi^ DC from retina during injury resolution appears unlikely to occur by lymphatic drainage, but their numbers decline by an unknown mechanism. Their decline in numbers suggests that the numbers of DiI^+^GFP^hi^ DC may be underestimated due to turnover. A few thousand GFP^hi^ DC appear, have the opportunity to acquire DiI^+^ debris, and disappear with their label over the course of two to three weeks, while the number of MG remains relatively constant.

T cell adaptive immune responses may contribute to neuroprotection. A number of neurodegenerative diseases/injuries are ‘sterile’, so that the antigens are limited to self-antigens. Nervous system self-antigen targets for immunopathogenic CNS autoimmune responses were reported to promote RGC survival post-ONC [47], perhaps by activated autoreactive T cells secreting neurotrophic factors promoting neuron survival [48]. Self-reactive Tregs were found to reduce RGC survival [23,24]. DC are the prototypical antigen presenting cells, and we showed elsewhere that retinal GFP^+^ DC present antigen to antigen-specific T cells [29], and that retinal DC upregulate MHC class II expression following ONC [21]. These properties may further link the role of retinal DC to T cells and influence neuroprotection.

Although infiltrating monocytic cells were the major source of TLR^+^ cells in traumatic brain injury [34], signaling pathways to NFκB activation in retina are not straightforward, a result that has been observed by others [49,50]. In preliminary studies, we found that the canonical signaling pathway via RelA is not well-used *in vivo* by mononuclear cells in murine retina (data not shown). Further, since the entire mouse shares in adaptor protein deficiency in these knockout mice, some outcomes may negate each other. This may account for results showing neuroprotection [46] or a lack of effect [51,52] in MyD88 and/or TRIF-deficient mice.

### A two-niche model for resident microglia and transient dendritic cells

Several observations suggest the presence of two overlapping niches in retina, the resident MG niche (N1) maintained at a relatively stable number, and a transient niche (N2) created by injury or inflammation that becomes occupied by recruited mononuclear cells, including GFP^hi^ DC (Figure 10). The finding that sublethal irradiation protects from glaucoma in DBA/2 J mice by reducing monocyte entry in the irradiated retina/ON [53] is consistent with a two-niche model. Retinal DC appear to occupy N2, distinct from MG/macrophages in N1. Relative to the GFP^hi^ DC in CDG retina, their recruitment in MTdko retina is substantially reduced (note Figure 6), but the GFP^hi^ cells that were recruited exhibited a similar ability to take up DiI-labeled RGC following an ONC. While the number of GFP^hi^ cells peaked at seven to thirteen days post-ONC followed by a rapid decline, moderately increased numbers persisted for at least two months post-ONC [21]. No definitive marker has been found to distinguish MG from DC, nor have factors that sustain N1 or N2 been identified. If these cells occupied the same niche, one would expect that both MG and the GFP^+^ cells would decline in proportion to their numbers, as the total number of cells returned to pre-injury levels. However, in the absence of catastrophic injury, N1 appears to continue to be occupied by MG, as allelic or other markers of recruited cells decline [54].

The chemokines and receptors that support recruitment of circulating precursors into multiple tissues, C-C chemokine receptor-2/C-C chemokine ligand-2 (CCR2/CCL2) and C-X3-C chemokine ligand-1; CX3CR1, C-X3-C chemokine receptor-1 (CX3CL1/CX3CR1) [55], do not appear to be strong candidates for mediating the recruitment, function or maintenance of the cells in retinal N1 [56], but may have more effect on N2. The effect of CX3CR1-deficiency on experimental autoimmune uveoretinitis (EAU) pathogenesis in mice was found to be insignificant by one lab [57], but was associated with more severe EAU by another [58]. Further, no evidence for effects on retinal development or injury repair was associated with CX3CR1 deficiency [59]. Evidence for differences in migration or tracking was found in mice deficient in both CCL2 and CX3CR1, but degenerative changes in the retina were not found [60]. Experimental autoimmune encephalomyelitis (EAE) was found to be more severe in CX3CR1-deficient mice [61], but in models for ischemia-reperfusion injury in kidney [62], spinal cord injury [63], and atherosclerosis [64], disease was less severe in CCR2^−/−^ or CX3CR1^−/−^ mice, and attributed to reduced myeloid cell recruitment. Knockout of either or both of these receptor/ligand pairs yields mice whose retinal myeloid cells bear some resemblance to the MTdko mice; that is the MG niche is largely intact, but recruitment is diminished. This is likely due to the role of NFkB in production of CX3CL1 on endothelial cells [65] stimulated by IL-1 and TNF [66]. Similarly, upregulation of CCR2 mediated by TLR2 and TLR4 ligation [67] would be diminished in MTdko mice, leading to reduced expression of accessory molecules and integrins associated with migration of cells into the retina [68–70]. Recent experiments with depletion protocols that may allow specific depletion of CNS MG may yield valuable information on the N1 niche [71], and strategies to manipulate N2.

## Conclusions

Use of MyD88 and/or TRIF deficient mice on the CDG background provided insights into the retinal injury response. Mice with single or double deficiencies in these adaptor proteins had reduced responses, based on finding fewer GFP^hi^ DC in injured retinas post-ONC. Although fewer in number, GFP^hi^ DC retained their ability to acquire DiI^+^ debris, suggesting that expression of these adaptor proteins, and their role in NFκB signaling, was not a factor in their ability to phagocytose dying RGC. Recruitment of GFP^hi^ DC, and their phagocytic activity, were distinct processes in which recruitment was diminished, but phagocytic activity was not. Instead, GFP^lo^ MG were more likely to become DiI^+^, suggesting that they filled the void left by the reduction in GFP^hi^ DC in MyD88/TRIF-deficient mice.

## Additional file

Additional file 1: Figure S1. Comparison of RGC counts/field obtained by manual and autocounting protocols. FG labeling combined with automated RGC counting was done to reduce observer bias. Field size = 0.190 mm^2^.

## Abbreviations

AMD: age-related macular degeneration
ANOVA: analysis of variance
B6: C57BL/6J
CCL2: C-C chemokine ligand-2
CCR2: C-C chemokine receptor-2
CDG: CD11c-DTR/GFP
CX3CL1: C-X3-C chemokine ligand-1
CX3CR1: C-X3-C chemokine receptor-1
DAPI: 4′,6-diamidino-2-phenylindole
DC: dendritic cell
DiI: di-alkyl-indocarbocyanine
DPBS: Dulbecco’s phosphate-buffered saline
DTR: diphtheria toxin receptor
DTx: diphtheria toxin
EAE: experimental autoimmune encephalomyelitis
EAU: experimental autoimmune uveoretinitis
FG: Fluorogold
GCL: ganglion cell layer
GFP: green fluorescent protein
IL-1: interleukin-1
IPL: inner plexiform layer
MG: microglia
MyD88: myeloid differentiation primary response gene (88)
Mko: MyD88 knockout
MTdko: MyD88/TRIF double knockout
NFL: nerve fiber layer
NFκB: nuclear factor kappa-light-chain-enhancer of activated B cells
ONC: optic nerve crush
PMN: polymorphonuclear granulocytes
RGC: retinal ganglion cell
TLR2: toll-like receptor 2
TLR4: toll-like receptor 4
TNF: tumor necrosis factor
TRIF: TIR-domain-containing adapter-inducing interferon-β
Tko: TRIF knockout
TUNEL: terminal deoxynucleotidyl transferase-mediated dUTP nick-end labeling
wt: wild type
