# Supplementary material for: Retinal dendritic cell recruitment, but not function, was inhibited in MyD88 and TRIF deficient mice
Source: J Neuroinflammation. 2014 Aug 13;11:143. doi: 10.1186/s12974-014-0143-1 (PMC4149240; doi:10.1186/s12974-014-0143-1)
Supplement: Additional file 1: Figure S1. — Comparison of RGC counts/field obtained by manual and autocounting protocols. FG labeling combined with automated RGC counting was done to reduce observer bias. Field size = 0.190 mm2. [file 12974_2014_143_MOESM1_ESM.docx]

Additional file 1


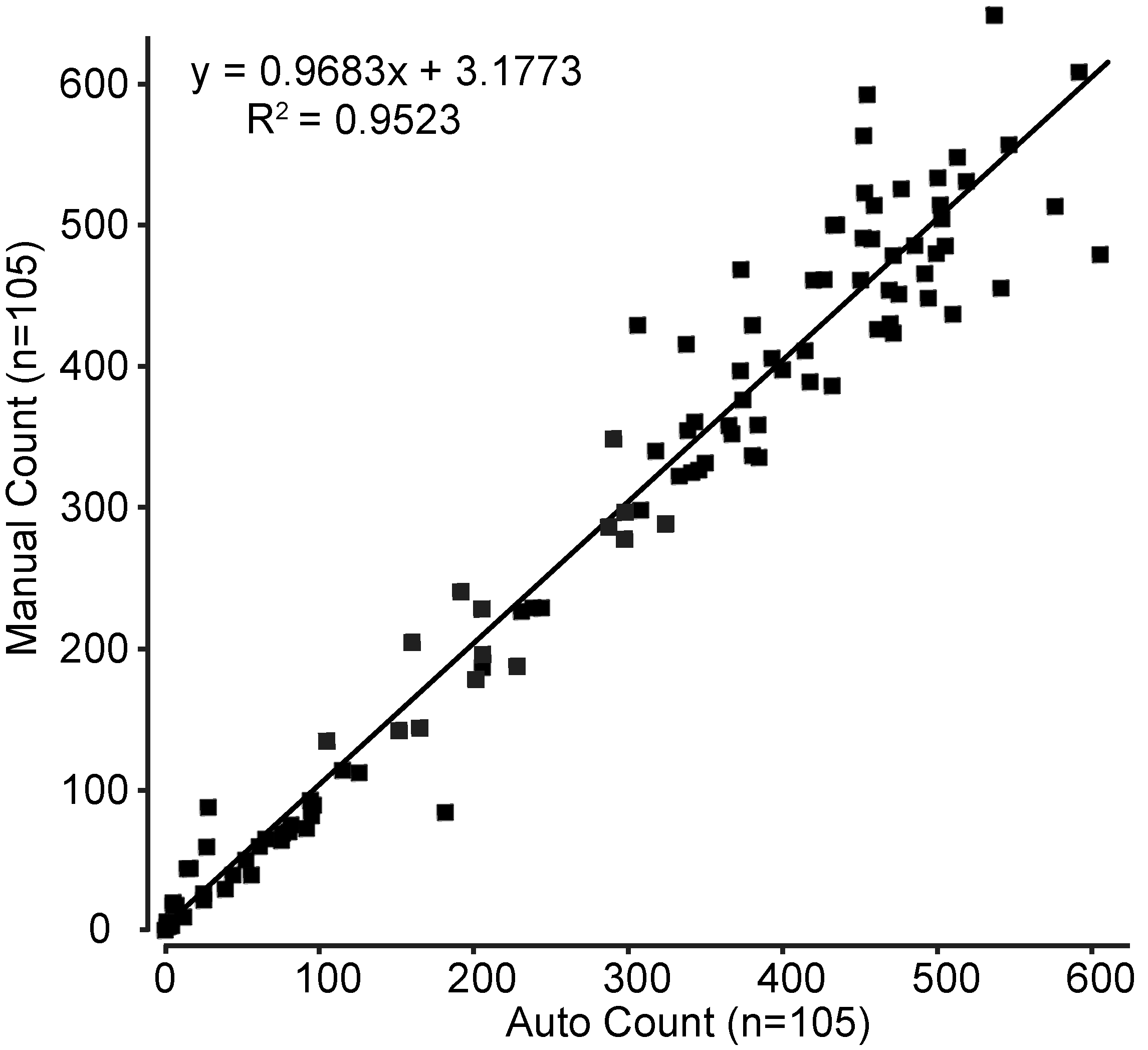


Supplement Fig. 1. Comparison of RGC counts/field obtained by manual and auto-counting protocols. FG labeling combined with automated RGC counting was done to reduce observer bias. Field size = 0.190 mm^2^.

Automated retinal ganglion cell (RGC) counting was used to show that the injury was present in the ONC mice. The automated protocol was confirmed in preliminary trials by comparison with manual counts (Supp. Fig. 1). The slope (0.97) and R^2^ (0.95) show both counting methods produced similar results.

***Automated RGC counting protocol***

Eight well-spaced non-overlapping regions of the retina were imaged and analyzed through high quality 20X RGB micrographs (Leica DM4000B fluorescence microscope with Leica DFC340FK camera). Due to variability in image brightness, all images were manually adjusted for brightness by a blinded observer before automated analysis in Adobe Photoshop and NIH ImageJ. In Adobe Photoshop, RGB images were grayscaled, processed through a high pass filter at 71.2 pixels and thresholded at 130. Thresholded images were inverted so that fluorescent particles appear black on a white background, and exported to Image J. Noise reduction was performed such that any particles less than 15 pixels were eliminated. Images were dilated twice to preserve circularity and watershed processing was performed to differentiate between particles that are touching each other. The resulting images were then analyzed via the Image J analyze tool. Particles ≥ 200 pixels were counted. Batched processing was performed on both the Adobe Photoshop and ImageJ portions of this process and the steps detailed above were algorithmically programmed on both software packages through the Batch Processing and Macros functions, respectively. As shown in Supp. Fig. 1, counts generated via this automated counting protocol are very highly correlated with manual counting performed by a blinded observer. The automated counting method was adapted from published reports [1, 2].

1. Nadal-Nicolas FM, Jimenez-Lopez M, Sobrado-Calvo P, Nieto-Lopez L, Canovas-Martinez I, Salinas-Navarro M, Vidal-Sanz M, Agudo M: **Brn3a as a marker of retinal ganglion cells: qualitative and quantitative time course studies in naive and optic nerve-injured retinas.** *Invest Ophthalmol Vis Sci* 2009, **50:**3860-3868.

2. Salinas-Navarro M, Jimenez-Lopez M, Valiente-Soriano FJ, Alarcon-Martinez L, Aviles-Trigueros M, Mayor S, Holmes T, Lund RD, Villegas-Perez MP, Vidal-Sanz M: **Retinal ganglion cell population in adult albino and pigmented mice: a computerized analysis of the entire population and its spatial distribution.** *Vision Res* 2009, **49:**637-647.
